# A case study of College English teachers' emotional experiences in the blended teaching context

**DOI:** 10.3389/fpsyg.2023.1303819

**Published:** 2023-12-29

**Authors:** Yu Shi, Haibo Gu, Qian Wang

**Affiliations:** ^1^School of Foreign Languages, Soochow University, Suzhou, China; ^2^Academy of Future Education, Xi'an-Jiaotong Liverpool University, Suzhou, China

**Keywords:** language teacher emotion, emotional experience, blended teaching, ecological theory, appraisal theory

## Abstract

The current research on language teacher education has witnessed a surge in studies focusing on teacher emotions due to their recognized importance in teaching. However, limited efforts have been found to investigate the emotions of Chinese College English teachers in the blended teaching context. This qualitative case study aims to uncover the emotional experiences of four Chinese College English teachers and explore the causes of their emotions in the blended teaching context. Data for the study was primarily gathered through interviews and case documents. The findings indicate that teachers in Eastern China experienced more positive emotions than negative emotions in the blended teaching context, while teachers in Western China exhibited the reverse pattern. These emotions were caused by their continuous appraisal of the interaction between their personal goals and various ecological systems. The research findings underscore the crucial role of teacher emotion in blended teaching and provide implications for enhancing blended teaching practices.

## 1 Introduction

Blended teaching has gained prominence in higher education due to technological advancements and the influence of COVID-19. Scholars have regarded it as the “new normal” in higher education (Dziuban et al., 2018; Jost et al., 2021), and also an inevitable trend in the reform of College English course in China, as it successfully breaks through time and space limitations, which is conducive to solving the problems of insufficient in-class hours and lack of language learning contexts outside the classroom in College English course. Characterized by the systematic integration of online and face-to-face learning, blended teaching leads to teachers' various emotional experiences, as they navigate between the online and offline worlds (Ellis, 2014; Zhao and Song, 2022). In the Chinese educational context, College English teachers grapple with challenges such as stress, burnout, diminished motivation, and job dissatisfaction, primarily stemming from factors like an overwhelming workload, the diminished status of College English courses, and reduced student motivation (Ding et al., 2022). Recognizing teaching as an inherently emotional practice (Hargreaves, 1998, 2000; Beatty, 2000), it is crucial to delve into the realm of teacher emotions. Such exploration holds profound implications for teaching quality, teacher-student relationships, professional development, and students' academic performance (Sutton and Wheatley, 2003; Schutz and Pekrun, 2007; Schutz and Zembylas, 2009). Understanding and managing teacher emotions are pivotal elements in creating a positive and effective learning environment. By cultivating the appropriate emotional states, English teachers can foster an atmosphere conducive to student success, active engagement, and enhanced overall language proficiency.

Teacher emotion is a socially constructed, personally enacted way of being that emerges from teachers' conscious and/or unconscious judgments regarding perceived successes at attaining goals or maintaining standards or beliefs during transactions as part of social-historical contexts (Schutz et al., 2006). It is constructed through their interactions with students, colleagues, administrators, and other parties in their respective pedagogical, institutional, and sociohistorical contexts (Ding et al., 2022). Previous studies have sought to investigate teacher emotion in the context of both offline teaching (e.g., Gkonou and Miller, 2021; Nazari et al., 2023) and online teaching (e.g., Gu et al., 2022; Liu et al., 2022, 2023; Wang and Song, 2022). Researchers found that although teachers in the offline teaching context shared some unpleasant emotional experiences (such as frustrations and anxiety), their dominant emotions were more positive than negative (Cross and Hong, 2012; Richards, 2022; Goetze, 2023). In the online teaching context, however, teachers experienced more negative emotions, such as anxiety and anger, than positive emotions, such as enjoyment and pride, due to students' failure to understand teachers' teaching goals (Wiebe and Kabata, 2010), low engagement in interactions (Liu et al., 2022), and relatively poor learning outcomes in online courses (Emerson and MacKay, 2011). In addition to student factors, features of online teaching, such as the high frequency of technological problems, difficulty in conducting interactions, and poor network conditions, are also crucial in eliciting various foreign language teacher emotions (Gu et al., 2022).

While research on teacher emotion has been done in the context of both offline teaching and online teaching, few concern teacher emotions in the blended teaching context. It is crucial to highlight the pressing necessity for an in-depth exploration of College English teachers' emotions within the blended teaching context. Their emotional experiences offer valuable insights into their perceptions of blended teaching. This investigation is pivotal for gaining a comprehensive understanding of the factors that may contribute to broader acceptance and the effective implementation of blended teaching in the post-pandemic era (Banihashem et al., 2023). Such insights are integral to improving the quality of College English course instruction. Additionally, it should be noted that since emotions are culturally specific (Mesquita et al., 2014), investigating emotions in the Chinese context allows for a nuanced understanding of how cultural elements may impact the emotional experiences of teachers. This understanding is vital for creating teaching strategies that resonate with the cultural context. Moreover, there is limited knowledge regarding the causes of teacher emotions in the blended teaching context. Understanding these causes is crucial for advancing the field beyond mere descriptions of emotional experiences and toward a comprehensive explanation of why particular emotions arise (Gu et al., 2022).

Thus, this study, adopting the qualitative case study method, is designed to unravel the emotional experiences of four Chinese College English teachers and to explore the causes of their emotions in the blended teaching context. In line with the purposes of the study, two research questions are addressed:

- RQ1. What emotions did these College English teachers experience in the blended teaching context?
- RQ2. What caused their emotions (e.g., enjoyment, pride, anger, and anxiety) in the blended teaching context?

This study serves the dual purpose of assisting College English teachers in comprehending their various emotional experiences within the blended teaching context and fostering reflection on the underlying causes of these emotions. Furthermore, the findings of this study offer administrators and school leaders valuable insights for refining blended teaching methodologies to accommodate the diverse emotional experiences of teachers.

## 2 Literature review

### 2.1 Teachers' perceptions of blended teaching

Blended teaching is an instructional method that combines both online and in-person instruction, and thus affords the learners the unique benefits of both modes of instruction (Dziuban et al., 2005; DeMolder et al., 2023). It is featured by increased flexibility and personalization, abundant learning resources, technical advantages (also accompanied by technical difficulties), and increased learning time (Zhao and Song, 2022). Combining face-to-face with technology-mediated instruction, blended teaching is considered the most effective and most popular mode of instruction adopted by educational institutions due to its perceived effectiveness in providing flexible, timely and continuous learning (Rasheed et al., 2020).

Previous studies have explored teachers' perceptions of blended teaching, shedding light on both its positive and negative effects. Banihashem et al. (2023) explored teachers' attitudes and emotions related to blended education and discovered that teachers faced high workloads and experienced stress due to challenges in work-life balance, course preparation, and technical issues. They also reported teachers' low wellbeing resulting from a lack of social and physical contact, along with difficulties in finding a balance between online and face-to-face education. However, despite these challenges, teachers demonstrated high motivation attributed to their personality, the flexibility of blended education, and technological advances. In another study, Wu and Luo (2022) investigated teachers' perceptions of blended learning and confirmed its positive impacts. They acknowledged its benefits of flexibility and accessibility, along with students' higher motivation, a better understanding of certain topics, and increased student-to-teacher interaction. Nevertheless, they admitted that blended teaching increased their time commitment to their jobs, and senior faculty faced technology challenges. Similarly, Zhao and Song (2021) found that teachers widely recognized blended learning but lacked confidence in implementing it effectively. Their top three difficulties included increased workload, insufficient funds for course development, and limited preparation time for online activities. Additionally, they expressed a clear need for pedagogical, financial, technical, and emotional support.

Overall, these studies reveal the complex nature of teachers' perceptions of blended teaching. While recognizing its advantages, such as increased motivation and interaction, flexibility and accessibility, teachers also face challenges related to workload, technology, and the need for support, which gives rise to teachers' different emotional experiences. Thus, this study focuses on exploring the emotional experiences of teachers in the blended teaching contexts and investigating how they are caused to address teachers' concerns and enhance teacher wellbeing and blended teaching effectiveness.

### 2.2 Teachers' emotional experiences in offline and online teaching context

Previous research has extensively examined teachers' emotional experiences in both offline and online teaching contexts. In offline teaching, Cross and Hong (2012) explored two elementary teachers' emotions within the school environment and found that although these teachers did encounter unpleasant emotional experiences such as disappointment and frustration, their dominant emotions were more positive than negative, and they were highly committed and satisfied in their careers. Being able to foreground pleasant emotions seemed to result from the teachers' ability to be empathetic and to refocus their thoughts and energies on the aspects of their environment they could change for the better. In the language teaching field, Gkonou and Miller (2021) explored how language teachers can move from emotional turmoil and important ethical dilemmas to emotional rewards. By analyzing critical incident stories, they found that although these experiences were initially cast as negative, upon reflection teachers framed the experiences as helping them learn how to deal with difficult students, to better understand students' behaviors and emotions, and to not view them as personal attacks. Teachers also treated these critical incidents as leading to positive and meaningful moments related to teaching. Teacher emotions could therefore be seen as being in a state of flux and changing within the same lesson, across lessons or even throughout one's career. Apart from the US and UK context, researchers in China have explored teacher emotion in the Chinese context. For example, Chen (2016) investigated the emotions experienced by primary teachers in Hong Kong and Mainland China schools and developed a five-factor Teacher Emotion Inventory (i.e., Joy, Love, Sadness, Anger, and Fear). Through surveying 254 teachers in a pilot study and 1830 teachers in the main study, she found that these teachers enjoyed positive interactions with students and colleagues, and recognition from school, family and the public, but experienced negative emotions due to unfair treatment, competition among colleagues, imbalance of work lives, and pressure from society, policy, and educational change.

More recently, online teaching has gained significant prominence and scholarly attention, particularly due to technological advancements and the post-epidemic era. Gu et al. (2022) explored the emotional experiences of five Chinese EFL teachers engaged in livestream teaching and found that teachers experienced a range of emotions, including predominant negative ones like anxiety, stress, and anger, alongside a few positive emotions such as satisfaction, love, and happiness. Some scholars have delved into specific emotions related to online teaching. For instance, Liu et al. (2022) investigated livestream English teaching anxiety experienced by 12 high school EFL teachers from China, identifying six types of anxiety related to factors at the macro, exo, and micro levels, including the COVID-19 pandemic, limited technological support from school authorities, students' parents, inadequate technological pedagogical and content knowledge, and insufficient effective teacher-student interactions. Additionally, there are some studies examining online teaching emotions indirectly. Wang and Song (2022) took interviews with a group of English teachers in China and found that their online teaching emotions were influenced by three main factors: the degree of adaptation to online teaching technology, the invisibility of the online teaching space, and the peripheral environment of the space in which the teachers were located. In addition, this study observed the online classrooms of the interviewees and found that the first two influences are at a priority level for Chinese English teachers, while whether the third factor can bring positive or negative emotions is often influenced by the nature of the emotions brought by the first two factors. In order to find a balance between norms and emotions, English language teachers engage in emotional labor in their work.

These studies collectively demonstrate that teachers experience diverse emotions, ranging from anxiety and anger to satisfaction and enjoyment, in both offline and online teaching contexts. However, there is a paucity of research dedicated to exploring the emotional experiences of Chinese College English teachers in the blended teaching context. Therefore, this study aims to address this gap by investigating the emotional experiences of Chinese College English teachers in the blended teaching environment, thereby offering valuable insights into the challenges, opportunities, and strategies associated with blended teaching, ultimately contributing to the understanding and improvement of teacher wellbeing and effectiveness in the rapidly evolving educational landscape.

### 2.3 Understanding teacher emotions from ecological and appraisal theories

With the “ecological turn” of applied linguistics (Larsen-Freeman, 2016), numerous studies (e.g., Cross and Hong, 2012; Liu et al., 2022) started to focus on language education from the perspective of ecology. Inspired by Bronfenbrenner's (1979) ecological system theory, a four-layered nested analytical framework is proposed to view the causes of teacher emotion. The microsystem contains various activities, an individual's roles and interpersonal relationships in a given setting with particular physical and material characteristics. In this study, the microsystem mainly consists of significant others (e.g., students and colleagues), and important activities (e.g., blended teaching model implementation), since all these are closely related to immediate experiences in blended teaching. The exosystem refers to a number of settings that do not actively involve the individual but affect, or are affected by, what happens in the setting containing the developing person. In this study, it includes school authorities and technicians. School authorities fulfill the responsibilities of implementing the requirements and policies from the local Education Bureau or the Ministry of Education (Liu et al., 2022), administrating the teachers' blended teaching and evaluating their teaching performance. In this system, teachers do not take part in making policies or requirements related to school management; however, these events may cause them various emotions. Technicians perform the duty to solve technological problems and maintain the online course platform to relieve teachers' technology anxiety. The macrosystem refers to the broader cultural context of an individual and influences interactions in all other layers. In this study, the macrosystem refers to the technology situation. The chronosystem represents the factor of time since time often brings changes in life events and experiences that alter the relationship between the individual and the environment.

In addition, Frenzel's (2014) reciprocal model on causes and effects of teacher emotion is also applied to explore the causes of teacher emotion. Teacher emotions result from appraisals pertaining to their personal goals. In specific, teachers' emotional experiences are determined based on their judgments regarding whether their personal goals are aligned with the triggering factors in the external environment. Regarding teachers' appraisals and resulting emotions, the model proposes that there are five important appraisal dimensions, including goal consistency appraisals, goal conduciveness appraisals, coping potential appraisals, goal attainment/impediment responsibility appraisals, and goal importance appraisals. Generally, goal consistency and goal conduciveness appraisals should determine the valence of the emotions experienced (positive vs. negative). Goal importance appraisals should determine the intensity of the emotional experience, with higher relevance typically leading to higher intensity for both positive and negative emotions. Coping potential and responsibility appraisals should determine the emotional valence and intensity, indicating the arousal or activation of emotions.

Enlightened by Bronfenbrenner's ecological system theory and Frenzel's model on causes of teacher emotions, this study proposed that teachers' emotions were caused by teachers' continuous appraisal between the interaction of their personal goals and ecological systems (i.e., microsystem, mesosystem and macrosystem) in their life. There were five types of appraisals in total and different appraisals combined elicited different emotions.

## 3 Methodology

A qualitative case study design was adopted in this study, which provides a detailed understanding of the way people make sense of phenomena in their context (Yin, 2018). This research approach allows for an in-depth, holistic examination of the experiences, perceptions, and emotions of teachers within the specific context of blended teaching.

### 3.1 Setting

The widespread adoption of information technology in Chinese colleges and universities has led to the popularization of network teaching platforms and autonomous learning platforms. Concurrently, College English teachers have been exploring new teaching models, such as online-offline blended teaching. This approach entails students attending offline classes in the classroom while utilizing online platforms as supplementary learning resources. Specifically, teachers share learning materials on the online platform prior to each class, requiring students to engage in pre-class online learning. Subsequently, students attend offline classes in the classroom. After each session, students participate in online discussions with their classmates and the teacher, sharing ideas and posing questions.

To ensure a comprehensive examination of the blended teaching context, this study was conducted in two cities: a well-developed city S located in the east of China, and a developing city W situated in the west of China. In city S, two universities were selected, including a comprehensive research university and a city university. In city W, a normal university that specializes in training teachers was chosen as the research site. This selection allows for a diverse representation of institutions, enabling a more extensive analysis of the emotional experiences of Chinese College English teachers in the blended teaching context.

### 3.2 Participants

In accordance with the criteria of “purposeful sampling” (Patton, 1990), four College English teachers were invited to participate in the study. With the purpose of effectively addressing research questions comprehensively and accurately, the participants were chosen due to their close association with the researcher and their willingness to share emotional experiences. Additionally, the selection process aimed to maximize variation among the participants by considering factors such as gender, age (*M* = 38.75, SD = 8.30), years of teaching experience (*M* = 12.25, *SD* = 9.00), academic title, educational background, and university type (see Table 1). The online and offline teaching hours of the four participants range from 12 to 18 h per week. This approach ensures comprehensive coverage of diverse situations pertaining to the phenomenon under investigation (Patton, 2015).

**Table 1:** Basic information of four participants.

**Participant**	**Gender**	**Age**	**Years of teaching**	**Professional title**	**Educational background**	**University type**
George	Male	37	7	Associate professor	Ph.D.	An east comprehensive university
Tina	Female	38	12	Lecturer	Master degree	An east city university
Zoe	Female	30	5	Lecturer	Master degree	A west normal university
Helen	Female	50	25	Associate professor	Master degree	A west normal university

### 3.3 Data collection

In this study, qualitative data was collected through semi-structured interviews and case documents to gain insights into the participants' emotional experiences within the context of blended teaching. The present study employed individual semi-structured interviews as its primary data source, a methodology previously utilized in the examination of teacher emotion (e.g., Miller and Gkonou, 2018). Semi-structured interviews, a common tool in qualitative research, strike a balance between the necessary structure for research consistency and the flexibility essential for a thorough exploration of participants' experiences. This approach was chosen to delve into the cognitive processes occurring within individuals' minds, particularly when direct observation is not feasible in diverse natural or social settings (Miles et al., 2014; Liu et al., 2022). Aligning with the growing recognition of the significance of informants' inner worlds (Mason, 2002), the interviews aimed to scrutinize teachers' emotional experiences and what caused their emotional experiences within the context of blended teaching. A total of four online interviews were conducted individually with each participant. Prior to each interview, the purpose of the study was reiterated, and the confidentiality of research data was assured. Participants willingly agreed to take part in the study. The interview questions consist of four sections exploring the teacher's background, the blended teaching model, teachers' emotional experiences and their causes in the blended teaching context.

All the interviews were conducted in Chinese, as using the first language of both the researcher and the participants helped better facilitate their expressions (Gu et al., 2022), which the participants confirmed. The pertinent findings were translated into English after data analysis. These interviews lasted ~60–90 min and were recorded with their consent using electronic recording devices. Detailed notes were taken during the interviews to capture participants' statements and expressions. In view of the ethical principles of research, all participants were given an interview invitation letter outlining the study, how data would be used as well as information on confidentiality and anonymity. Participants signed the accompanying consent form and filled out the bio-data sheet before being interviewed. In the interest of the participants, all names, specific places and other possible identifying information have been anonymized (Hofstadler et al., 2021).

Case documents, including teachers' blended teaching materials and chat records with the researcher, served as supplementary data sources to enhance the trustworthiness and authenticity of the interview data. Specifically, teachers' blended teaching materials encompassed teaching syllabi and PowerPoint presentations (PPTs) detailing the design of blended teaching. Teachers' teaching syllabi offered concrete information about the teaching schedule, weekly teaching content, and teaching goals, enriching the researcher's understanding of the blended teaching model adopted in their classes and providing insights into the teacher's teaching goals. Additionally, some teachers shared their PPTs, explaining how and why they implemented the blended teaching model in their College English classes. Moreover, the researchers established personal communication with the participants through WeChat, a widely used social communication app in China. This type of communication helped establish rapport between the researcher and participants, enabling a comfortable environment for sharing personally meaningful stories and feelings. In addition, it served as a valuable tool for verifying interview content in instances where information was unclear.

### 3.4 Data analysis

The inductive approach was deployed in the qualitative data analysis. The recorded interviews were first transcribed into four transcripts by using IFLYREC Series Voice-to-Text Products (https://www.iflyrec.com/), with a total word count of over 120,000. Following that, the transcripts were carefully checked and proofread by the authors and the four participants. Revisions were made when errors or misinterpretations were identified during the transcription process. During the initial reading, the entire transcripts were reviewed to identify specific episodes related to teacher emotions, indicated by keywords such as “pride,” “anxiety,” and “anger,” along with their respective causes.

In subsequent readings, content analysis was applied to explore deeper meanings within the interview data, facilitating a comprehensive and nuanced interpretation. Episodes were assigned into different categories and sub-categories. Consequently, themes regarding the causes of teacher emotions were identified. For example, anger was found to be caused by (a) interactions between teachers' goals and students' behaviors in the microsystem, (b) interactions between teachers' goals and technology situations in the macrosystem, and (c) interactions between teachers' goals and school authorities in the exosystem. Following that, sub-categories were further divided from these categories. For instance, students' cheating on their homework, students' ignorance of online learning resources, and students' inattentive listening to teachers in class were attributed to the subordinate category of “interactions between teachers' goals and students' behaviors.” To ensure the trustworthiness of the data analysis, member checks were conducted during the study. After completing the first draft of the coding categories, the first and second researchers independently coded the transcriptions. Later, two coding tables were double-checked to add, elicit, or integrate any information, forming a more complete and reliable coding scheme (Gu et al., 2022).

## 4 Findings

The findings below represent different emotions experienced by the participants in this study, as well as their respective antecedents. With regard to the first research question, the teachers reported a total of seven types of emotions, including enjoyment, pride, anger, anxiety, helplessness, disappointment and gratitude (listed in order of frequency). Concerning the second research question, only the most frequently experienced emotions were presented. Data analysis revealed that four emotions, enjoyment, pride, anger and anxiety, were mentioned far more frequently than any other emotions by the participants. Moreover, considering that enjoyment, anger, and anxiety are the three basic emotions which have been found to be most salient and most frequent among teachers (see Sutton and Wheatley, 2003; Frenzel, 2014), and pride is also commonly experienced by teachers perhaps second to enjoyment (Frenzel, 2014), this study attempted to examine the causes of these four emotions by combining ecological with appraisal theories.

### 4.1 College English teachers' emotional experiences in the blended teaching context

This section addresses the first research question on various types of emotional experiences of four College English teachers in the blended teaching context. The teachers reported a total of seven types of emotions, comprising three positive ones, namely, enjoyment, pride, and gratitude (listed in order of frequency), and four negative emotions, namely, anger, anxiety, helplessness, and disappointment.

#### 4.1.1 Positive emotions

The participants reported experiencing the following three emotions: enjoyment (with a frequency count of 16), pride (with a frequency count of 11), and gratitude (with a frequency count of 3).

##### 4.1.1.1 Enjoyment

First, the most frequently reported positive emotion among the teachers was enjoyment. Three major types of enjoyment emerged in the order of frequency: enjoyment related to the advantages of the blended teaching model implementation, enjoyment related to students' positive behaviors, and enjoyment related to colleagues' praises. All four teachers acknowledged the sense of enjoyment they derived from the advantages of implementing the blended teaching model, such as making up for the limited offline learning time, increasing learning resources, providing convenience for students and teachers, and enhancing teacher-student interactions. For example, Tina expressed her enjoyment when discussing how the blended teaching model resolved the issue of limited learning time for students:

**Extract 1**.

*One significant advantage of the blended teaching model is that we, as teachers, can share a wealth of learning resources on the online course platform. As we know, the class time allocated for College English has been compressed, while our teaching content is not reduced. Thanks to the blended teaching model, knowledge that cannot be covered in the classroom can now be uploaded to the online course platform for students to access after class. Consequently, it helps us address the challenge of insufficient amount of offline class time for College English. I felt very happy when it contributed to resolving this issue. (Tina, Interview, 12 Dec. 2022)*

George and Zoe also derived enjoyment from their students' positive behaviors. Both of them expressed happiness upon students' active interactions and the delivery of high-quality homework. Furthermore, George experienced additional enjoyment when receiving acknowledgment from his colleagues:

**Extract 2**.

*When I first started my teaching career, my colleagues told me that they were very satisfied with the course I designed, and my students also liked it very much. This made me feel very happy, and it seemed that my colleagues were quite confident in my performance as a newcomer. (George, Interview, 10 Feb. 2023)*

##### 4.1.1.2 Pride

All four participants experienced pride in the blended teaching context, including pride related to students' positive behaviors, colleagues' praises, school authorities' recognition and leading colleagues in exploring blended teaching. The primary source of pride for these teachers was the positive behaviors exhibited by their students, including benefiting from the class, expressing appreciation for the teacher, and providing positive feedback. Zoe, for example, felt proud when her students enjoyed and benefited from her class:

**Extract 3**.

*I've had a few classes where some outgoing students took the initiative to send unsolicited WeChat messages after class, telling me how much they enjoyed and were inspired by my teaching. I experienced pride when I saw those messages, and I think this is the meaning of being a teacher. (Zoe, Interview, 12 Feb. 2023)*

Beyond student-related aspects, teachers also took pride in earning praise from colleagues and recognition from school authorities. For instance, Tina took pride in the recognition she received for developing an online platform for the College English course. Her colleagues actively used and appreciated its various functions, which significantly affected her emotions. She has also gained recognition from school leadership and was encouraged to apply for a prestigious designation. This recognition brought her immense pride and highlighted the value her course provided to a diverse student body. The approval and support from school leaders further contributed to her sense of pride.

Helen experienced pride when she led her colleagues in exploring blended teaching implementation in the initial stage:

**Extract 4**.

*In 2016, I initiated the implementation of blended teaching in the College English department and took on a leadership role in guiding my colleagues. Drawing inspiration from peer schools and studying relevant literature, I developed a comprehensive plan for blended teaching. This included creating examples such as sample learning task designs and short video micro-lessons, which I used to provide training to my fellow teachers. I felt a sense of pride in being a pioneer during that time. (Helen, Interview, 13 Feb. 2023)*

##### 4.1.1.3 Gratitude

Two participants, George and Tina, experienced altogether three kinds of gratitude, including gratitude related to school authorities' support, students' assistance, and colleagues' cooperation. George expressed his gratitude for receiving emotional support from school authorities and assistance from his students. The vice dean of his college strongly supported education reform and encouraged him to explore the innovative blended teaching model. Beyond school authorities' support, George also received assistance from students who volunteered as teacher assistants, aiding in the search for online learning materials and enriching the online platform.

Tina, while discussing her online course development experience, expressed gratitude for her colleagues' active participation and valuable assistance. Despite facing pressure, Tina's collaborative team divided tasks efficiently, ensuring the successful completion of all assigned responsibilities:

**Extract 5**.

*During the process of developing online courses, I experienced tremendous pressure, but I am grateful to have an excellent team of teachers. Each teacher in the team actively participated in course development and helped me with a lot of work. For instance, we formed groups, with two teachers responsible for listening and another two for reading and translation writing. We worked collaboratively as a team, rather than me fighting alone. My team is exceptional, and together we successfully completed all tasks. (Tina, Interview, 12 Dec. 2022)*

#### 4.1.2 Negative emotions

A total of four negative emotions were reported by the participants. In order of frequency, the emotions are as follows: anger (with a frequency count of 9), anxiety (with a frequency count of 9), helplessness (with a frequency count of 6) and disappointment (with a frequency count of 5).

##### 4.1.2.1 Anger

Three major types of anger emerged in the order of frequency: anger related to technology failure, anger related to students' misbehavior, and anger related to school authorities' unreasonable arrangements. Technology failure, such as the unstable network and the breakdown of the online course platform, was the primary factor that elicited the teachers' anger. Zoe, who is a College English teacher in Western China, said the following:

**Extract 6**.

*I remember once I had a class in the classroom and needed to use the online course platform resources, but I ended up getting angry because the computer in our school responded slowly and there were problems logging in to the online platform, plus the network was stuck. I wasted at least ten minutes waiting to switch networks, and I felt that these technological problems restricted me, and the pace of my class was completely disrupted, and both students and I felt really awful. (Zoe, Interview, 12 Feb. 2023)*

Apart from technological challenges, teachers also felt angry due to students' misbehavior, such as cheating during online tests and ignoring online learning resources. Helen, for instance, experienced anger triggered by her students' dishonesty. During the interview, she detailed the practice of assigning tests on the online course platform every 2 weeks, typically comprised of objective questions due to the large student population. On one occasion, she was astonished and dismayed to find that some students had completed all 50 questions within a minute. Helen, expressing disbelief and ire, questioned how they could achieve such speed and accuracy. Confronting the students, she underscored the significance of honesty and genuine effort, discouraging deceptive practices and expressing her anger.

Another teacher, George, shared a similar source of anger related to students' misconduct. Embracing blended teaching, he dedicated much effort to providing abundant learning resources on the online course platform. However, he discovered that some students opted to disregard these resources rather than make use of them. George, feeling exasperated, openly expressed his anger to his students for ~10 min, marking it as the first instance of losing his temper.

In a different context, Zoe voiced her anger regarding what she perceived as unreasonable arrangements by school authorities:

**Extract 7**.

*The school administration's implementation of the blended teaching model seemed exaggerated to me. They viewed it as challenging and insisted on providing training in advance, a measure I found unnecessary. For example, when it came to utilizing Rain Classroom (an online learning platform), they mandated training sessions. Initially anticipating difficulties, we discovered that the platform was quite straightforward after the training, yet it consumed at least two hours of our time. This situation was not only frustrating but also infuriating, as it felt like an essentially time-wasting endeavor. (Zoe, Interview, 12 Feb. 2023)*

##### 4.1.2.2 Anxiety

All four participants experienced anxiety in the blended teaching context, which includes anxiety related to the disadvantages of blended teaching and students' negative performance. Three teachers, Tina, George and Helen, experienced anxiety due to the disadvantages of blended teaching, including increased workload, frequently-occurred technological problems (e.g., online platform breakdown) and online resources building pressure. For instance, Tina felt anxious and worried that she might make language mistakes in front of a large crowd of students:

**Extract 8**.

*I was responsible for building the learning resources on the online course platform, and the precision of language use was highly required, so I felt very anxious and worried about making mistakes. Because my online course was open to 42 classes with more than 2,000 freshmen in the whole school, I felt anxious because even if I made a mistake in one single word, it would have a huge impact at the school level and impose overwhelming psychological pressure on me. (Tina, Interview, 12 Dec. 2022)*

Zoe experienced anxiety related to students' poor performance. She emphasized the significance of student initiative and self-discipline. She expressed concern about the poor performance of some students in College English class, due to their lack of active participation in class interactions. As an elective course teacher, Zoe strived to make the class engaging and interesting but failed to attract some students' attention.

##### 4.1.2.3 Helplessness

Tina, George and Helen experienced three kinds of helplessness, including helplessness related to the school's lack of technological support, students' negative performance and lack of reference to the blended teaching model implementation.

For example, Helen expressed her helplessness related to students' poor performance. She highlighted that some students lacked self-discipline, often failing to meet deadlines for pre-learning tasks and providing excuses. As a teacher, she tended to grant extensions and avoided setting time limits on online platforms. This compromise, though not ideal, was out of helplessness:

**Extract 9**.

*Sometimes, our students lacked self-discipline and self-restraint. For example, when given a week to complete an online pre-learning task, many students failed to meet the deadline and provided excuses like “Sorry, we had too many assignments” or “I forgot about the deadline, can I have an extension?” As a teacher, I often gave in to these requests out of helplessness and abandoned setting time limits on online platforms, except for the final grade submission deadline. While this approach was not ideal, it felt like a necessary compromise. (Helen, Interview, 13 Feb. 2023)*

Tina felt helpless due to the lack of reference to the blended teaching model implementation:

**Extract 10**.

*During the implementation of blended teaching, I encountered feelings of helplessness. Since everyone was still figuring out this innovative teaching model, there were no definitive answers available. I had to solve problems on my own by researching and discussing with teachers. Unlike traditional classroom teaching, where experienced teachers can offer valuable advice, blended teaching is unfamiliar to everyone. Therefore, it is in its early stages as a new concept, and this led to moments of helplessness. (Tina, Interview, 12 Dec. 2022)*

##### 4.1.2.4 Disappointment

George, Helen, and Zoe experienced three kinds of disappointment in total, including disappointment related to students' negative behaviors, colleagues' indifferent attitudes and challenges of blended teaching. Helen expressed her disappointment with certain colleagues who prioritized completing tasks over quality in blended teaching. Their performance often fell short of her expectations:

**Extract 11**.

*Among my colleagues engaged in blended teaching, there's a segment that seems less involved, prioritizing task completion over refining the quality of instruction. In an effort to inspire higher standards, I initiated a blended teaching demonstration, hoping it would set a benchmark. Unfortunately, the outcomes they presented occasionally didn't match my anticipated standards. This consistent gap between expectations and reality has been a source of ongoing disappointment throughout the blended teaching implementation. Despite this, I've come to a compromise and accepted this existing scenario. (Helen, Interview, 13 Feb. 2023)*

Helen also expressed her disappointment concerning the practical effects of the blended teaching model implementation:

**Extract 12**.

*I am disappointed that the blended teaching model implementation was not as effective as it should have been. As a matter of fact, students here in W city have a busy schedule with numerous classes and activities throughout the week. Their limited free time makes it challenging for them to complete pre-learning tasks for all their subjects. This puts significant pressure on my students, and as a teacher, I understand their struggles. I gradually accepted that sometimes they are unable to meet my expectations, which can be disappointing. However, I am also making efforts to improve the efficiency of in-class teaching, aiming to save some of their pre-learning time and allow them more time to focus on their core subjects. (Helen, Interview, 13 Feb. 2023)*

### 4.2 Causes of College English teachers' emotions in the blended teaching context

The findings revealed that teacher emotions were caused by the appraisals teachers made regarding their individual goals within the blended teaching context. More specifically, teachers actively pursued specific key goals in the blended teaching context and continuously made judgments pertaining to these goals based on their perceptions of the external environment. This external environment encompassed the microsystem, exosystem, and macrosystem, ultimately giving rise to diverse emotional experiences among teachers.

#### 4.2.1 Causes of positive emotions

##### 4.2.1.1 Causes of enjoyment: positive goal attainment

The findings showed that enjoyment was caused by appraisals related to positive goal attainment. Data analysis in this study revealed that enjoyment arose from the interplay between teachers' personal goals and microsystems. The microsystem factors that elicited enjoyment include the implementation of a blended teaching model, student behaviors, and colleague behaviors.

**Figure 1:**
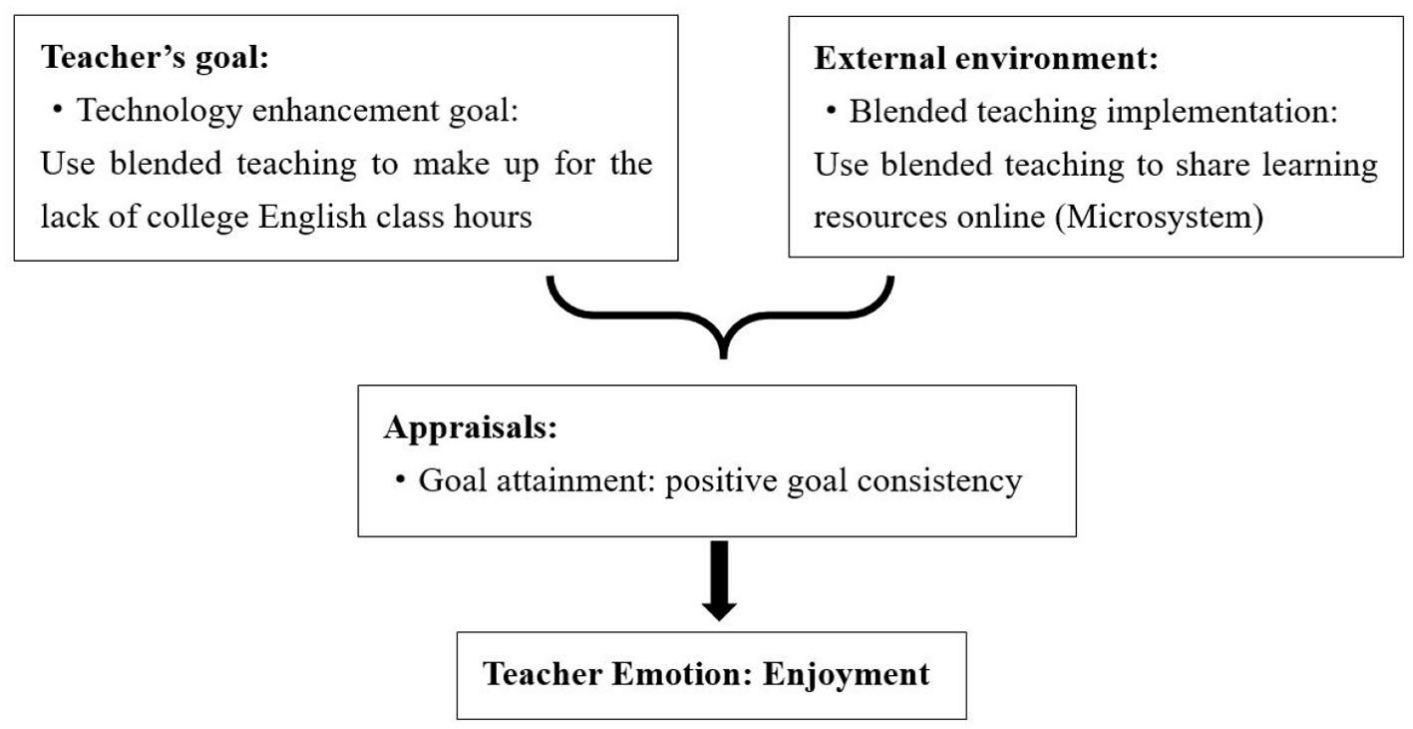
Causes of Tina's enjoyment.

First, the benefits derived from the implementation of the blended teaching model, including heightened flexibility, increased convenience, and an augmented array of learning resources, aligned with teachers' individual goals such as improving teaching effectiveness. Consequently, they evoke a sense of enjoyment among teachers. For example, Tina shared in the interview that the blended teaching model allowed teachers to share learning resources on an online platform, making up for compressed class time. It helped address the challenge of limited offline class hours for College English course, contributing to resolving the issue, giving rise to her enjoyment (see Figure 1).

**Figure 2:**
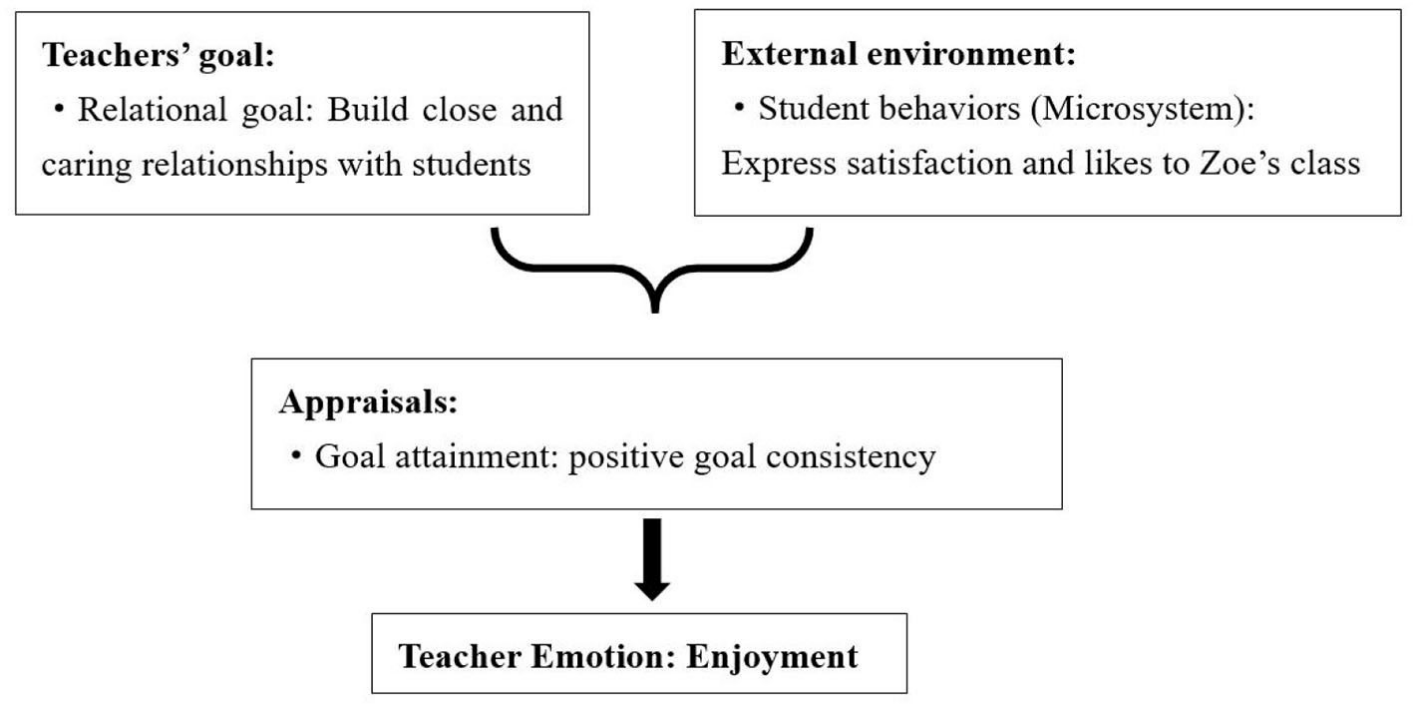
Causes of Zoe's enjoyment.

Additionally, enjoyment can be derived from the alignment of teachers' personal goals and students' positive behaviors. Zoe, for example, experienced joy as her students actively expressed their satisfaction with her class, aligning with her goal of establishing strong student-teacher relationships (Figure 2). Similarly, George found enjoyment when his students showcased substantial learning in their after-class reflective assignments, reflecting positive engagement and motivation tied to his goal of fostering student development.

**Figure 3:**
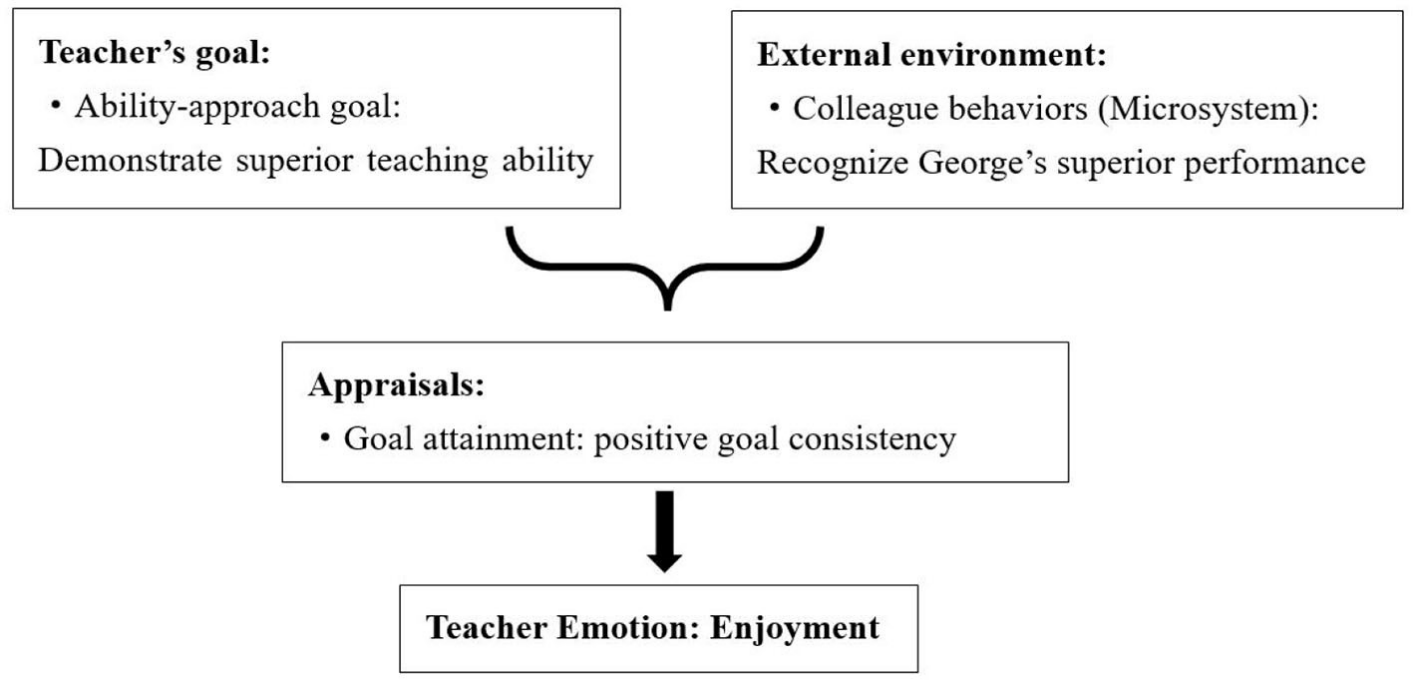
Causes of George's enjoyment.

Furthermore, George's happiness extended to receiving praise from his colleagues, fulfilling his aspiration for recognition (Figure 3). George shared, “My colleagues expressed satisfaction with my course design, and my students appreciated it. It brings me joy to witness my colleagues maintaining a positive perception of my work as a newcomer in the profession.” This external validation resonated with George's goal, contributing to his overall sense of enjoyment.

##### 4.2.1.2 Causes of pride: positive goal attainment and internal goal attainment responsibility

Pride arose from positive goal attainment and internal goal attainment responsibility, as revealed in the study's data analysis. The interplay between teachers' personal goals and microsystems, along with exosystems, contributed to this sense of pride. In the microsystem, students' positive behaviors and colleagues' recognition served as key antecedents, while school authorities' support played a role in the exosystem.

**Figure 4:**
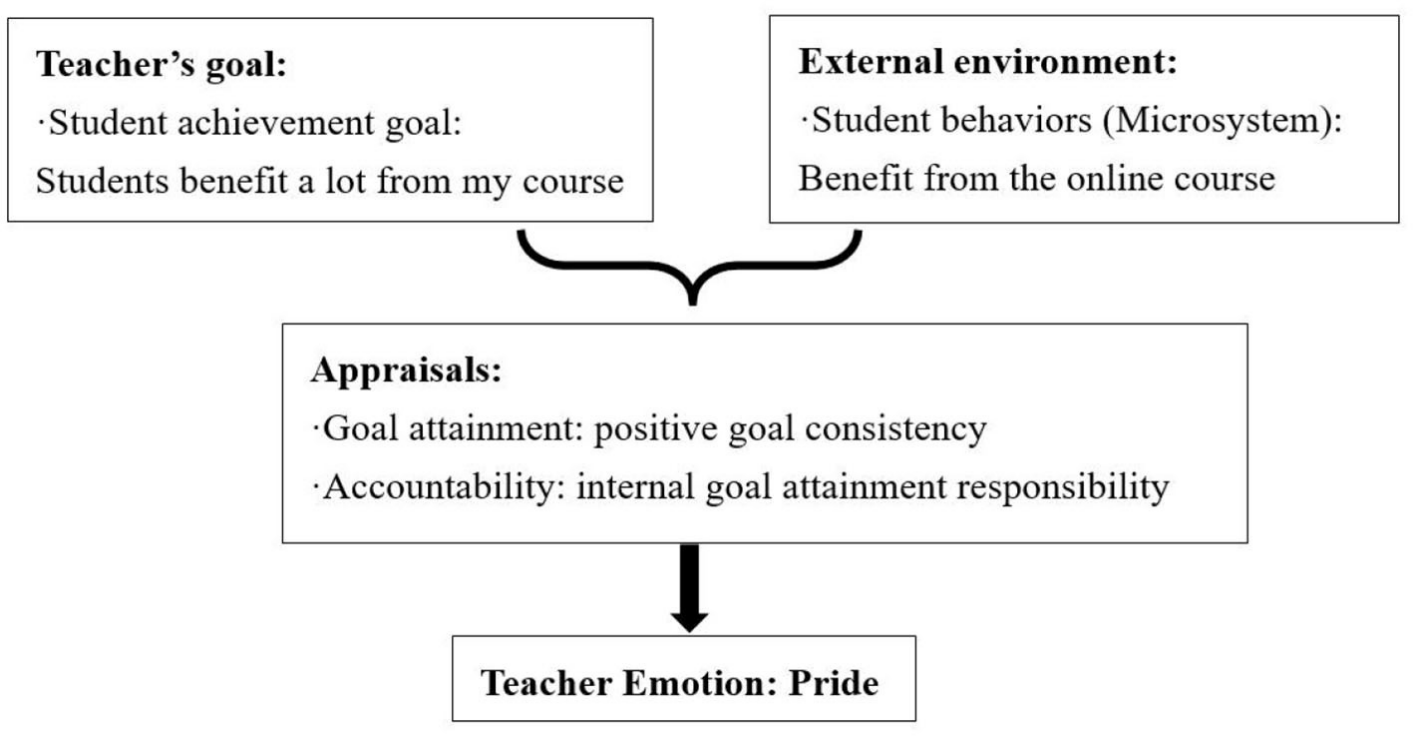
Causes of Tina's pride.

Teachers experienced pride when they evaluated that students' positive behaviors aligned with their personal goals and attributed this success to internal goal attainment responsibility. Tina exemplified this in her online classes, expressing overwhelming pride when her teaching brought tangible benefits to students (see Figure 4):

**Extract 13**.

*When my online classes do bring some benefits to the students, my pride is overwhelming, because my definition of pride lies in ensuring that students gain significant value from my teachings. To me, true success is measured by the extent to which students truly benefit from their learning experience. (Tina, Interview, 12 Dec. 2022)*

In the exosystem, Tina experienced pride when her course was praised and valued by school authorities, who also approved her application for a first-class course at the school level. The recognition from school authorities was consistent with Tina's goal, and Tina mainly attributed it to her personal hard work, thus eliciting pride:

**Extract 14**.

*Being entrusted with the responsibility for the College English course has garnered recognition from school leadership. School leaders have even encouraged me to apply for the prestigious “First-Class Course” designation at the institutional level. This recognition brings me immense pride. It not only signifies that students benefit from my course but also underscores the value the leadership places on my course, which caters to a diverse student body. Their approval and support in endorsing my project further contribute to my sense of pride. (Tina, Interview, December 12, 2022)*

#### 4.2.2 Causes of negative emotions

##### 4.2.2.1 Causes of anger: negative goal attainment and external goal impediment responsibility

Anger was caused by negative goal attainment and external goal impediment responsibility. The analysis of the data in this study showed that anger emerged from the interaction of teachers' personal goals and unfavorable technology situations in macrosystems, students' negative behaviors in microsystems, as well as school authorities' unreasonable arrangements in exosystems. When teachers appraised that those antecedents in the external environment were inconsistent with their personal goals, and they attributed it to external goal impediment responsibility, anger emerged.

Unfavorable technology situations in macrosystems, which mainly refer to unstable network connections and the breakdown of online platforms, were the primary external sources of anger. During a technical failure, three teachers, George, Helen, and Zoe, committed to enhancing teaching quality, found themselves grappling with anger. Their anger stemmed from the disruptive impact of technology failure, an incongruence with their shared goal of elevating the quality of education. This emotional response was further intensified as they attributed the setback to an external factor, labeling it as a hindrance beyond their control. The dual nature of this experience, both as a technical obstacle and an external goal impediment, amplified their emotional response, resulting in a sense of anger.

Two teachers, George and Helen, experienced anger as a result of students' misbehaviors in microsystems. George diligently prepared extensive learning resources on the online course platform, hoping that students would engage with them and study effectively. However, he discovered that the students did not read the resources at all, leading to his anger. In this scenario, George's goal of engaging students with online learning resources seemed to be incongruent with the students' passive behavior, causing him to attribute the impediment of his goal to the students, and thus experience anger.

**Figure 5:**
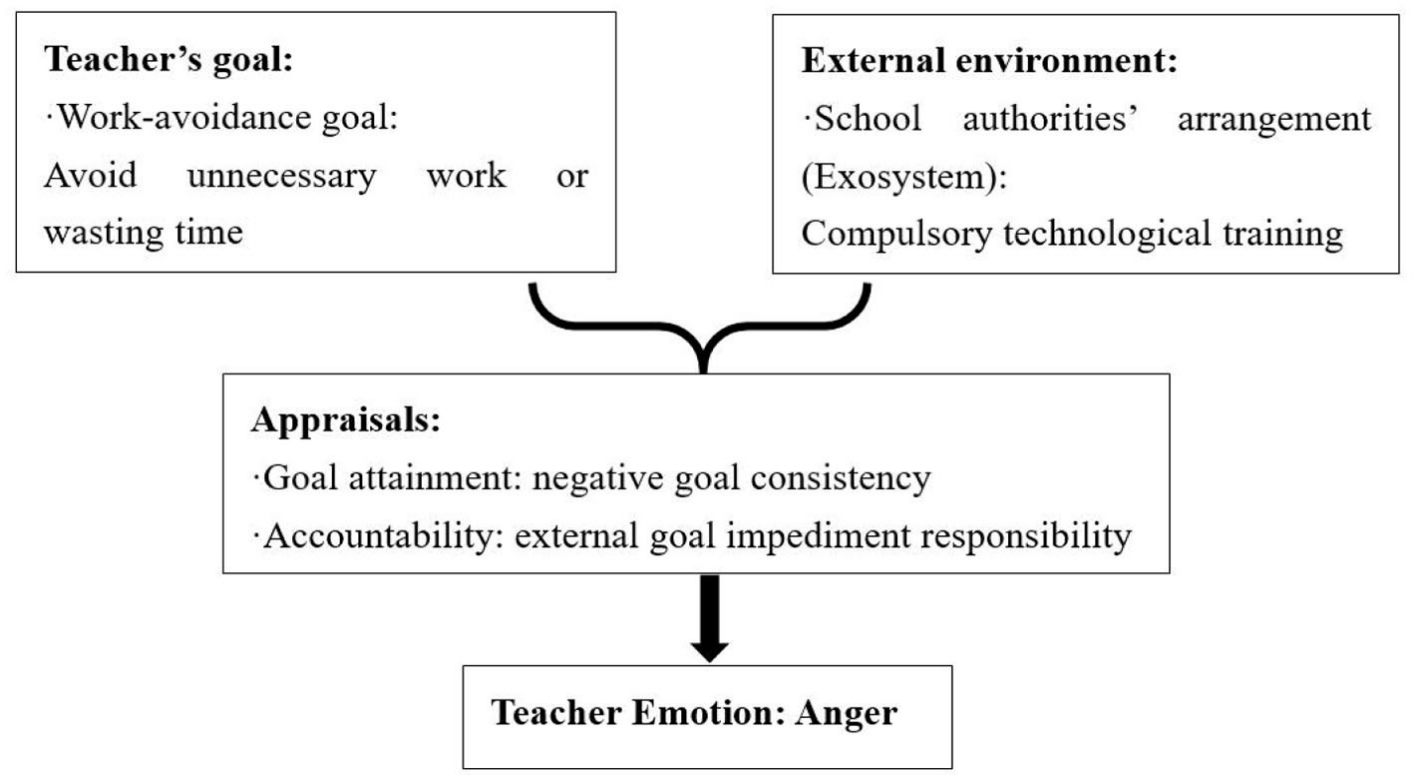
Causes of Zoe's anger.

One teacher, Zoe, felt angry due to school authorities' unreasonable arrangements in the exosystem (see Figure 5). She was compelled by school authorities to undergo training for blended teaching models, which she considered unnecessary and a waste of time. Despite her strong reluctance, she had to comply with the school's directive. Consequently, Zoe experienced anger stemming from negative goal attainment and external responsibility for impeding her goals.

##### 4.2.2.2 Causes of anxiety: negative goal attainment, low coping potential and internal goal impediment responsibility

Anxiety was caused by negative goal consistency or conduciveness, teachers' low coping potential, and internal goal impediment responsibility. The analysis of the data showed that anxiety emerged from the interaction of teachers' personal goals, disadvantages of blended teaching model implementation, and students' negative behaviors in microsystems.

**Figure 6:**
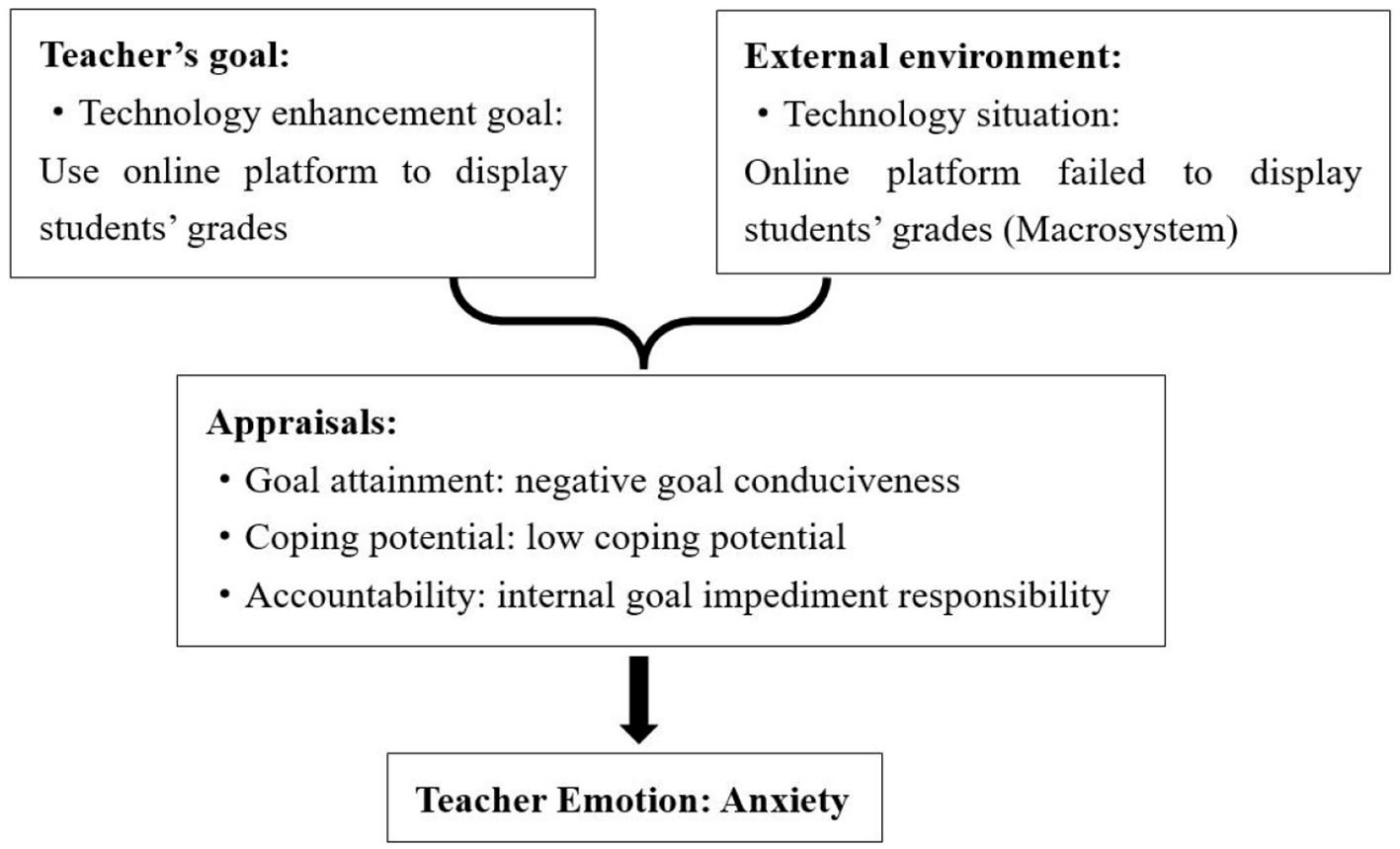
Causes of Helen's anxiety.

The disadvantages of blended teaching model implementation, which include increased workload and technological problems, were the primary triggering factors of anxiety. In this study, three teachers, George, Helen, and Tina, experienced anxiety during the implementation of the blended teaching model. Helen specifically expressed her anxiety due to her limited capability to handle technological issues. She recalled an instance where the online platform failed to display students' grades, leaving her unsure of how to resolve the problem. This resulted in negative goal attainment and a low coping potential for Helen. Furthermore, she held herself responsible for being unable to address technological challenges, intensifying her internal goal impediment responsibility and contributing to her anxiety (see Figure 6).

**Figure 7:**
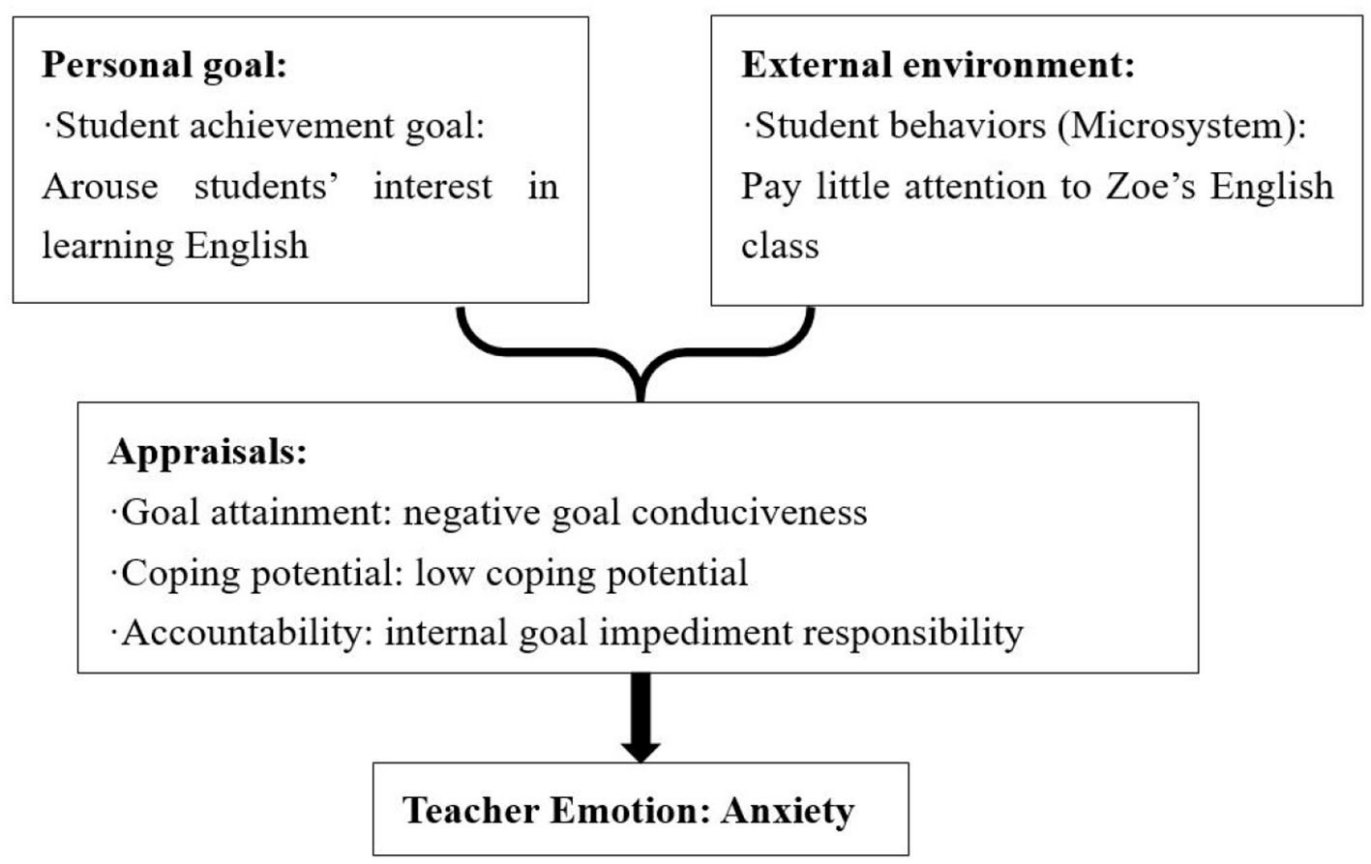
Causes of Zoe's anxiety.

Zoe felt anxious about her students' English learning because they paid little attention to the teacher in her class, which was inconsistent with her goal (see Figure 7). She struggled with finding ways to engage her students and lacked confidence in capturing their interest. Zoe's low coping potential and self-blame for her inability to captivate her students further fueled her anxiety.

## 5 Discussion

### 5.1 Highlighting important factors that shape teachers' emotional experiences in the blended teaching context

In this study, we have attempted to explore College English teachers' emotional experiences and their causes in the blended teaching context. The study's findings revealed that College English teachers in Eastern China generally experienced more positive emotions than negative emotions in the blended teaching context, while teachers in Western China exhibited the opposite pattern (see Table 2). This aligns with previous research indicating that teachers' emotions are culturally specific (Mesquita et al., 2014). The sociocultural disparities between the eastern and western regions of China, such as students' digital literacy, school's financial support and teachers' attitudes toward technology, contribute to the divergent emotional experiences of teachers in these areas.

**Table 2:** Teachers' emotional experiences with frequency counts in Eastern and Western China.

**Themes/sub-themes**	**Teachers in Eastern China**	**Teachers in Western China**
Positive emotions (30)	19	11
Enjoyment (16)	8	8
Pride (11)	8	3
Gratitude (3)	3	0
Negative emotions (29)	14	15
Anger (9)	3	6
Anxiety (9)	6	3
Helplessness (6)	3	3
Disappointment (5)	2	3

Three significant factors shape teachers' emotional experiences in the blended teaching context, including the features of blended teaching, technology and students' behaviors.

First, it is important to acknowledge that the features of the blended teaching model play a crucial role in shaping teachers' emotions in the blended teaching context. Enjoyment was the most frequently experienced emotion by teachers in both Eastern and Western China. The primary contributing factor to their enjoyment was the implementation advantages of the blended teaching model, such as compensating for limited offline learning time, expanding learning resources, offering convenience and flexibility for students and teachers, and fostering teacher-student interactions. Additionally, this study identified that anxiety, the most commonly experienced negative emotion by teachers in Eastern China, was mainly elicited by the disadvantages associated with the blended teaching model's implementation. These drawbacks include increased workload for teachers, technological difficulties or problems, and low fault tolerance for online language resources, etc. Notably, the advantages and disadvantages reported by participants in this study align with the typical features of the blended teaching model (Halverson and Graham, 2019; Banihashem et al., 2023), which include enhanced flexibility and personalization, increased interaction opportunities, technical advantages, abundant learning resources, and a high workload and stress due to challenges in work-life balance, course preparation, and technical issues. Furthermore, this study uncovered additional features of the blended teaching model. Teachers may experience heightened anxieties due to concerns about making language mistakes when creating online resources. Moreover, teachers are required to enhance their technological skills to address frequently occurring technological issues.

Second, this study reaffirms findings from previous research indicating that teachers' relationship with technology plays a key role in them feeling positive or negative emotions (Badia et al., 2019), which is quite understandable in a blended teaching context. Data analysis revealed that technology failure was the primary cause of anger, the most frequently experienced emotion among teachers in West China. In addition, the findings suggested that receiving support in dealing with technological problems and technology development can alleviate teachers' anxieties. Specifically, teachers initially experienced anxiety due to frequent technological issues, but their concerns gradually diminished as they received timely assistance from technicians, technological support from school authorities, and witnessed improvements in online platforms. These findings underscore the importance of providing adequate technological support to teachers, aligning with prior research (Gu et al., 2022) which suggests that teachers' positive emotions emerged when institutions provided more support for them, for example, by giving more technology training. However, it should be noted that rigid and inadequate technology training may cause negative emotions among teachers.

Third, students serve as a significant predictor of teacher emotions. It is important to note that compared with traditional face-to-face learning, students in the blended teaching context require more preparation time before class. In addition, blended teaching calls for a high level of self-regulation, presenting a considerable challenge for students with poor self-regulation skills (Van Laer and Elen, 2017; Zhao and Song, 2022). However, data analysis demonstrated that some students from West China often struggled with self-regulation, frequently breaking the deadline for submitting their assignments, giving rise to teachers' helplessness. Another pressing issue is that some students from remote rural areas in West China are reported to have low digital literacy and limited prior experience in using technology. In consequence, they encounter great difficulties in adapting to the blended teaching model, leading to teachers' anxieties and helplessness. Nevertheless, some students from East China are reported to exhibit high levels of digital literacy and aid teachers in resolving technological problems. Consequently, the teachers share their sense of gratitude with their students for their proactive assistance. This underscores the crucial role of self-regulation in blended teaching (Zhao and Song, 2022). Students with higher levels of self-regulation are better equipped to adapt to new circumstances and offer support to teachers when facing technological challenges. However, it also points out the need to address the digital divide and provide adequate resources and support to students from regions with limited access to technology, empowering them to improve their digital literacy and enhance their self-regulation skills.

### 5.2 Understanding how teacher emotions were caused from ecological and appraisal theories

This empirical study suggested that a combination of ecological and appraisal theories is conducive to understanding the causes of teacher emotion. As shown in this study, the causes of teachers' emotions involve the interaction between teachers' internal personal goals and external ecological environments. Specifically, the ecological environments of these College English teachers consist of four layers, including microsystem, exosystem, macrosystem, and chronosystem. By viewing teacher emotions through the prism of the chronosystem it may be surmised that teachers' emotions may change over time, due to changes in life events and experiences brought by time that alter the relationship between the individual and the environment. Taking anxiety as an example, Tina, a College English teacher in Eastern China, felt anxious at the initial stage of the blended teaching model implementation, while her anxiety gradually decreased since she got more familiar with coping with technology problems, and she felt relieved due to the online platform improvements and its functional refinements, as well as technicians' support. This is resonant with previous reports that teacher anxiety is an interactive, emotional and temporal psychological factor caused by the complex interactions between teachers and physical and virtue ecosystems at micro, exo and macro levels (Liu et al., 2022).

Moreover, this study suggested that adopting an appraisal-based approach is conducive to comprehending the emergence of teacher emotions. It furnished empirical evidence to illuminate how College English teachers can sustain and enhance positive emotions while mitigating negative ones within the Chinese context. Teachers are encouraged to deploy cognitive reappraisal techniques, reinterpreting adverse factors in their external environment to foster positive shifts in their emotional responses. Specifically, when confronted with the demanding task of collecting comprehensive learning resources, George chose to perceive it as an opportunity to inspire students and hone his skills. Consequently, he not only transformed potential anxiety into a sense of enjoyment but also contributed positively to the teaching-learning dynamic. Similarly, Helen pragmatically adjusted her goals to align with the nature of College English as a non-major course, recognizing the competing demands on students' time with their major subjects. As a result, she gradually accepted the fact that students occasionally missed deadlines, and she successfully managed her emotions by tempering her expectations, thereby alleviating any frustration or anger that might arise from unmet standards. This adaptive approach contributes to a more positive and harmonious teaching experience for both teachers and students alike.

Confirming Frenzel's (2014) study, our findings showcased that teacher emotions result from appraisals pertaining to their personal goals, and different appraisals combined may lead to different emotions. Taking anger and anxiety as an example, teacher anger was mainly linked to external causes such as technology failure and student misbehaviors, whereas anxiety was mainly linked to internal causes, such as the teachers' inability or lack of preparation to cope with the disadvantages of blended teaching model implementation, or inability to arouse students' interest in their class. These findings echoed Goetze (2023) in that the appraisals of accountability may play a role in differentiating the type of teachers' (negative) emotions. A self-directed focus resulted in anxiety, while an other-directed focus elicited anger.

## 6 Conclusions and implications

This paper examined the emotions of four Chinese College English teachers in the blended teaching context using ecological and appraisal theories. The findings revealed that College English teachers in Eastern China generally experienced more positive emotions, while teachers in Western China exhibited a different pattern with more negative emotions. The study also uncovered the causes of College English teachers' emotions in this context, showing that emotions are elicited based on teachers' appraisals of their personal goals and their (in)congruence with the external environment. Different combinations of appraisals result in varying emotional experiences: enjoyment is caused by positive goal attainment, pride is caused by positive goal attainment and internal goal responsibility, anger is caused by negative goal attainment and external goal impediments, and anxiety is caused by goal consistency or conduciveness, low coping potential, and internal goal impediments. This research contributes to our understanding of the causes of teacher emotions in the blended teaching context.

Nevertheless, the study is not without limitations. First, given that the data sources were restricted to interview data and case documents, it is hoped that future research could diversify the data sources by including offline classroom observations and recorded online classroom videos. Second, the study solely relied on one single interview per participant, limiting the depth and breadth of information gathered. However, conducting longitudinal studies and tracking interviews in future research can offer valuable insights into the dynamic nature of emotional experiences and their causes over time. Third, this study focused only on the causes of the four most frequent and typical emotions: enjoyment, pride, anger, and anxiety. To gain a comprehensive understanding, future studies could explore additional emotions such as disappointment, sadness and gratitude.

Despite the aforementioned limitations, this study provides the following implications at personal, micro, exo, and macro levels regarding ways to cope with College English teachers' emotional experiences and enhance blended teaching practices. At the personal level, teachers are encouraged to seize opportunities for developing their technology-enhanced instructional skills to effectively navigate potential challenges associated with technology integration. At the micro level, providing students with proper guidance and supervision is crucial in fostering their positive behavior and cultivating a cooperative learning environment. At the exo level, school administrators should not only offer financial and technological support to teachers but also empower them with increased autonomy in implementing the blended teaching model. At the macro level, technicians should take into account teachers' goals and needs when developing platform functionalities while ensuring platform stability and minimizing disruptions.

## Data availability statement

The original contributions presented in the study are included in the article/supplementary material, further inquiries can be directed to the corresponding authors.

## Ethics statement

The studies involving human participants were reviewed and approved by the Graduate School, Soochow University. The patients/participants provided their written informed consent to participate in this study.

## Author contributions

YS: Writing – original draft, Writing – review & editing. HG: Writing – review & editing, Supervision. QW: Writing – review & editing.
